# Comparative effects of deep brain stimulation in subthalamic nucleus and globus pallidus interna on verbal fluency and working memory in adult populations with parkinson’s disease: A systematic review

**DOI:** 10.1016/j.prdoa.2025.100355

**Published:** 2025-06-09

**Authors:** Hannah Trotman, Benjamin Jelley, Katja Umla-Runge

**Affiliations:** Cardiff University, School of Medicine, United Kingdom

**Keywords:** Parkinson’s disease, Deep brain stimulation, Verbal fluency, Working memory, Subthalamic nucleus, Globus pallidus interna, Cognitive function

## Abstract

**Background:**

Deep Brain Stimulation (DBS) in the subthalamic nucleus and globus pallidus interna is a well-established treatment for motor symptoms in Parkinson’s Disease (PD). However, the cognitive effects of DBS, particularly on verbal fluency and working memory, remain less clear.

**Purpose:**

This systematic review explores the comparative effects of subthalamic nucleus and globus pallidus interna DBS on verbal fluency and working memory in adults with PD, addressing gaps in current cognitive outcome data.

**Methods:**

A comprehensive search of EMBASE, MEDLINE, EMCARE, and PsycINFO was conducted. Studies were selected based on predefined criteria, focusing on randomised and non-randomised controlled trials involving adult PD patients treated with subthalamic nucleus or globus pallidus interna DBS. Data extraction and risk of bias assessments were performed.

**Results:**

Eight studies were included, with varied findings. Most studies observed a decline in verbal fluency following DBS, with no significant differences between subthalamic nucleus and globus pallidus interna targets. Working memory outcomes were also mixed; however, one study showed a statistically significant result favouring globus pallidus interna DBS for working memory.

**Conclusions:**

The cognitive effects of DBS appear variable and target-independent, highlighting the need for individualised treatment planning. While DBS effectively addresses motor symptoms, its cognitive impacts, especially on verbal fluency and working memory, require further exploration. These findings support a more personalised DBS approach, considering cognitive profiles, implantation laterality, and long-term outcomes.

## Highlights

• Verbal fluency often declines after deep brain stimulation in Parkinson’s patients.
• Working memory outcomes show more variability after subthalamic stimulation.
• One study found better memory outcomes after globus pallidus interna stimulation.
• Right-side brain stimulation may help protect verbal fluency in some patients.
• Findings support tailoring brain stimulation to reduce cognitive side effects.

## 1 Introduction

### 1.1 Background

Parkinson's Disease (PD) is a neurodegenerative disorder primarily affecting motor function due to the degeneration of dopamine-producing neurons in the substantia nigra. This loss leads to the hallmark motor symptoms of PD, including tremor, rigidity, bradykinesia, and postural instability. However, PD also has significant non-motor symptoms, such as cognitive decline, which greatly affect patients' quality of life [1,2].

Deep Brain Stimulation (DBS) has emerged as a revolutionary treatment for managing motor symptoms in PD, especially in patients who no longer respond adequately to pharmacological treatments [3,4]. DBS involves the implantation of electrodes in specific brain regions, typically the subthalamic nucleus or the globus pallidus interna (GPi), to modulate neural activity and restore motor function. While the motor benefits of DBS are well-documented, its effects on cognitive functions, particularly verbal fluency and working memory, remain less clear and are subject to ongoing research [5,6].

Verbal fluency and working memory are critical cognitive domains that are often affected in PD. Verbal fluency, which refers to the ability to generate words based on phonemic or semantic cues, is essential for communication and social interaction. Impairments in verbal fluency can lead to significant difficulties in daily communication, impacting social relationships and overall quality of life [7]. Working memory, the cognitive system responsible for temporarily holding and manipulating information, is crucial for tasks such as language comprehension, problem-solving, and planning [8]. Working memory deficits in PD can lead to challenges in carrying out complex cognitive tasks and hinder patients' ability to function independently [9].

Subthalamic nucleus and GPi DBS, while both effective for motor management, may differentially affect cognition due to their involvement in distinct neural circuits [10,11]. There is ongoing debate in the literature regarding the cognitive safety of subthalamic nucleus DBS versus GPi-DBS. Subthalamic nucleus DBS has often been perceived as riskier in terms of cognitive decline, particularly verbal fluency; however, emerging findings suggest that this dogma may be outdated and not consistently supported by empirical evidence [12,13].

Understanding the cognitive impacts of DBS is crucial for optimising patient care. Identifying which patients are at higher risk for post-operative cognitive decline could lead to more personalised DBS target selection and the incorporation of cognitive rehabilitation strategies [14]. This review aims to address these gaps by systematically examining the effects of subthalamic nucleus and GPi DBS on verbal fluency and working memory, contributing valuable insights to the field of neuromodulation in PD.

### 1.2 Aims

The primary aim of this research is to systematically investigate the effects of DBS in the subthalamic nucleus and GPi on verbal fluency in adult patients with PD. Given the critical role of verbal fluency in communication and quality of life, understanding how DBS impacts this cognitive function is essential for guiding therapeutic decisions.

The secondary aim is to examine the impact of DBS in the subthalamic nucleus and GPi on working memory, a fundamental domain of executive functioning. By assessing these outcomes, the review aims to build a clearer picture of how target site selection may influence cognitive trajectories post-DBS.

### 1.3 Hypothesis

It is hypothesised that subthalamic nucleus DBS would be associated with greater declines in verbal fluency compared to baseline and to GPi DBS, while GPi DBS would show more favourable or stable cognitive profiles, particularly in working memory outcomes.

## 2 Methods

The PICOS [15] framework was adopted to formulate the review question (Table 1).

**Table 1:** PICOS Framework.

**PICOS framework**	**Relation to systematic review**
**Patient**	• Adult population
	• Patients diagnosed with Parkinson's Disease
**Intervention**	• Deep brain stimulation
**Comparison**	• DBS in Subthalamic Nucleus
	• DBS in Globus Pallidus interna
**Outcome**	• Changes in verbal fluency
	• Changes in working memory
**Study**	• Comparative studies

### 2.1 Search Strategy

EMBASE, MEDLINE, EMCARE, and PsycINFO databases were searched for the final time on 10th July 2023. The results of these searches can be found in appendix A.

To conduct a comprehensive scope of literature, hand searches of reference lists were conducted. This yielded a further 2 studies for consideration. Moreover, google scholar and Grey Literature were also searched, yielding a further 5 studies for consideration.

### 2.2 Study inclusion criteria

Only primary research studies with rigorous methodologies were included. This encompassed randomised controlled trials (RCTs), non-randomised control trials (NRCTs), and prospective and retrospective cohort studies. The inclusion of studies with these robust designs ensured the reliability and validity of the systematic review by limiting bias.

Studies were included if they: included adult participants (aged 18 + ) with a clinical diagnosis of Parkinson’s Disease; involved participants who underwent DBS targeting either the subthalamic nucleus or the globus pallidus interna; reported pre- and post-operative assessments using standardised, quantitative measures of verbal fluency and/or working memory; and conducted a direct comparison between the two stimulation targets.

Studies were excluded if they: focused on a single target only without comparison; lacked cognitive outcome data; did not include pre-operative baseline assessments; were reviews, editorials, protocols, or case reports; or, involved experimental stimulation targets not relevant to this review.

There were no restrictions on gender or ethnicity.

### 2.3 Study Limits and exclusions

The FDA approved the use of DBS at the subthalamic nucleus or GPi in 2002 and therefore the literature search was initiated from the year 2002 [16]. The reason for this was that following the FDA approval copious amounts of research and clinical investigations specifically focused on understanding the cognitive improvements associated with DBS. Research prior to 2002 focuses primarily on the thalamus [17]. Therefore, due to the aim of this review focusing on modern day applications of DBS, using literature from a period in which subthalamic nucleus and GPi were not recognised as therapeutic options may yield irrelevant and outdated sources.

Articles were limited to English language and studies with human participants only. Studies that did not clearly specify the type or location of DBS electrode placement were excluded to ensure the inclusion of studies that specifically focus on the research question at hand.

Studies that involved interventions or treatments other than DBS for PD were excluded to ensure a focused analysis specifically on the effects of subthalamic nucleus and GPi DBS. Studies investigating other interventions, or a combination of interventions, would be excluded to maintain the specificity of the research question.

### 2.4 Study selection process

A two-stage screening approach for the studies was employed. Initially, titles and abstracts were screened based on the inclusion and exclusion criteria. Studies that passed this initial screening then underwent full-text assessment for final inclusion. The screening process was performed by a single researcher [18].

### 2.5 Quality assessment and data extraction

#### 2.5.1 Data extraction

The data extraction process was conducted meticulously to gather comprehensive and relevant information from the selected studies. A structured data extraction form was developed, incorporating predefined fields to systematically capture key data elements across the included studies [see supplemental materials 1].

#### 2.5.2 Risk of bias

A quality assessment of the included studies was performed to evaluate the methodological rigor and potential sources of bias.

The decision to utilise the Cochrane Risk of Bias Visualisation (ROBVIS) tool [19] for assessing the risk of bias in RCTs was driven by its distinctive graphical approach and its advantages over other available tools (e.g., Jadad Scale, Cochrane Risk of Bias Tool).

Similarly, the choice of the Risk of Bias in Non-randomised Studies − of Interventions (ROBINS-I) tool for assessing bias in NRCTs was guided by its suitability for non-randomised designs and its distinct advantages compared to other options. While tools like the Newcastle-Ottawa Scale (NOS) [20] and the Downs and Black Scale [21] exist for bias assessment in NRCTs, ROBINS-I provides a comprehensive framework specifically designed for interventions.

### 2.6 Data synthesis and analysis

A meta-analysis was initially considered. However, this approach was deemed inappropriate due to several critical factors: substantial heterogeneity in outcome measures used across studies (e.g., differing verbal fluency tests, varied working memory tests); variability in follow-up durations and assessment time points (ranging from 6 months to 3 years); inconsistent statistical reporting, including missing standard deviations and unclear effect sizes; and diverse study designs (e.g., prospective vs retrospective).

Combining such disparate data in a meta-analysis may have introduced significant bias and reduced the interpretability of pooled estimates. Therefore, the Synthesis Without Meta-analysis (SWiM) framework [22] was applied to guide a transparent and structured narrative synthesis. This allowed for the systematic organisation of findings while respecting the methodological diversity of included studies.

## 3 Results

Results of this systematic review will be presented in two main subsections, ‘verbal fluency’ and ‘working memory’.

### 3.1 Selection process

**Fig. 1:**
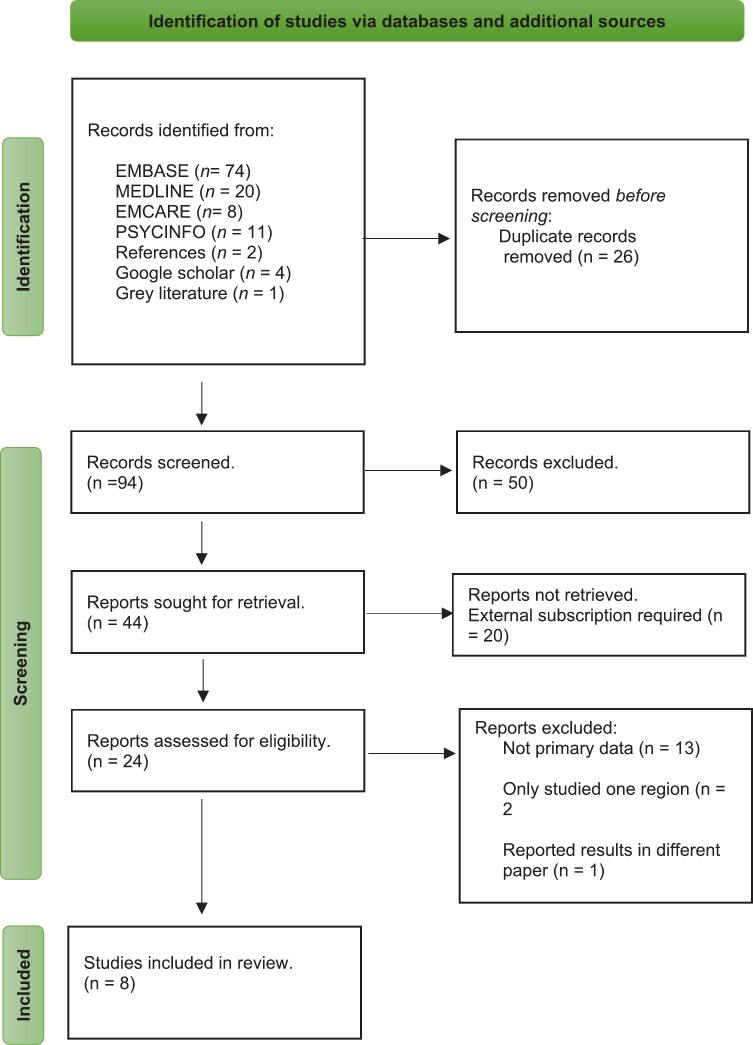
Flow Diagram based on PRISMA guidelines [23].

The selection process adhered to PRISMA guidelines [23]. From an initial pool of 94 records, duplicates were removed, and titles and abstracts were screened, resulting in 44 reports sought for full-text retrieval. Ultimately, 8 studies were included in the review after excluding ineligible studies based on full-text assessment. Fig. 1 displays the PRISMA flow chart, providing a comprehensive overview of the study selection process.

### 3.2 Data extraction

There were noticeable patterns and variations in the demographic details of participants in the studies. Across the included studies, the average age of participants generally fell within the early sixties, though some variation was observed. A few studies reported younger or older group means, but these were not systematically linked to stimulation target. Gender distribution was consistently skewed towards males, reflecting the known higher prevalence of Parkinson’s Disease in men [1]. Sample sizes varied considerably across studies, ranging from small cohorts of under 10 participants to larger trials with over 100 participants per group.

Table 2 displays each study in accordance with the data extraction form mentioned in the methods section.

**Table 2:** Study and participant characteristics.

**Study**	**Design**	**Country**	**Average age (years)**		**Gender (male:female)**		**Subgroups N=**	**N=**	**verbal fluency measure**	**Working memory measure**	**Results**	**Statistical test outcome**
			**GPi**	**subthalamic nucleus**	**GPi**	**subthalamic nucleus**						
**Alley 2022**	Nonrandomised controlled trial	USA	57.2 62.6		2:3 3:1		subthalamic nucleus = 3 GPi = 6	9	DKEFS Controlled Oral Word Association Test	N/A	Greater decline in subthalamic nucleus vs GPi	Nonsignificant
**Boel et al. 2016**	RCT	Netherlands	59.1 60.9		44:19 44:21		subthalamic nucleus = 65 GPi = 63	128	Controlled Oral Word Association Test, phenomic and category	N/A	Equal decline in GPi and subthalamic nucleus DBS	Nonsignificant
**Follet et al. 2010**	RCT	USA	61.8 61.9		113:19 116:31		subthalamic nucleus = 147 GPi = 152	299	Boston Naming Test Animal naming test	Wechsler Adult Intelligence scale working memory index	Neurocognitive outcomes were better in GPi DBS group than subthalamic nucleus DBS group	Nonsignificant
**Hansen et al. 2017**	Retrospective cohort	USA	66.7 64.8		9:3 11:6		subthalamic nucleus = 17 GPi = 12	29	Boston naming test	Working Memory Index	GPi patients showed no difference on any neuropsychological test. subthalamic nucleus scored lower	Nonsignificant
**John et al. 2021**	Nonrandomised controlled trial	USA	63.03 53.34		11:6 18:5		subthalamic nucleus = 23 GPi = 17	40	DKEFS Verbal Fluency	N/A	Verbal fluency decline equally in both subthalamic nucleus and GPi	Nonsignificant
**Okun et al. 2009**	RCT	USA	60.2 59.8		16:7 15:7		subthalamic nucleus = 22 GPi = 23	52	Letter fluency, semantic fluency	N/A	Worse performance on letter fluency tasks following subthalamic nucleus DBS No changes on the semantic fluency task	Nonsignificant
**Rothlind et al. 2007**	RCT	USA	60.2 61.4		18:5 15:4		subthalamic nucleus = 19 GPi = 23	42	Controlled Oral Word Association Test	WAIS-R DSST, WAIS-R	Equal decline in GPi and subthalamic nucleus DBS	Nonsignificant
**Weaver et al. 2012**	RCT	USA	60.4 60.7		77:12 56:14		subthalamic nucleus = 70 GPi = 89	159	The Hopkins Verbal Learning Test	Brief Visuospatial Memory Test	GPi slight worsening at 6 months, subthalamic nucleus worsened by 36 months	Working memory significant Verbal fluency nonsignificant

### 3.3 Risk of bias and data synthesis

#### 3.3.1 Risk of bias

**Fig. 2:**
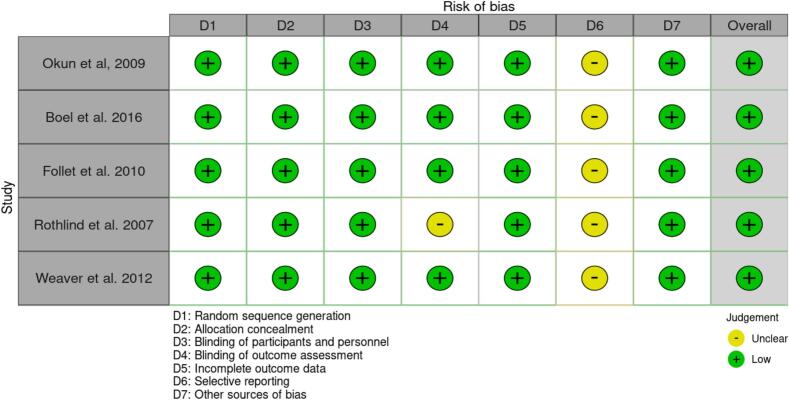
ROBVIS for RCT.

Fig. 2 displays that the overall risk of bias in each of the RCTs was relatively low. However, some studies had unclear reporting on selective reporting bias and blinding procedures [29]. The implications of these limitations on the overall quality and reliability of the evidence should be considered when interpreting the findings of the systematic review.

Despite the identified uncertainties, the overall risk was low. This judgement considers the comprehensive evaluation of multiple domains of bias. Although it is still important to interpret the findings with caution, the overall low risk of bias provides confidence in the reliability of the evidence presented in this systematic review.

**Fig. 3:**
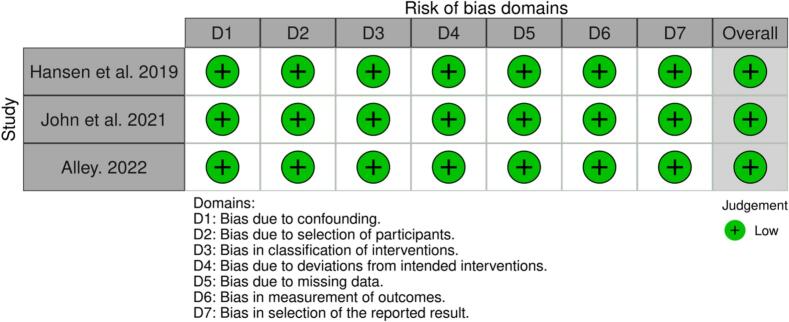
ROBINS-I for NRCTs.

Fig. 3 visually displays the completed ROBINS-I for the remaining 3 NRCTs. Hansen et al. [26], John et al. [25], and Alley [24] all exhibited an overall low risk of bias in the ROBINS-I assessment due to their rigorous methodology.

### 3.4 Data synthesis

Table 3 displays results for all included studies.

**Table 3:** Results of included studies.

**Study**	**Sample size per subgroup**	**Statistical test**	**Assessment measure**	**subthalamic nucleus DBS**		**GPi DBS**		**GPi VS subthalamic nucleus P value**
				Baseline Mean (SD)	Follow-up Mean (SD)	Baseline Mean (SD)	Follow-up Mean (SD)	
**Alley (2022)**	subthalamic nucleus = 3	ANOVA	Phonemic fluency b	13	6.5	11.6	9.8	NS
				*−1.9*	*−1.7*	*−1.7*	*−1.6*	*P* = 0.94
	GPi = 6							
		ANOVA	Semantic fluency b	11	7.8	12.2	9.8	NS
				*−2.2*	*−1.3*	*−1.9*	*−1.2*	*P* = 0.94
							
		T-Test	Action fluency b	9	//	6.8	//	//
				*−2.9*	*(//)*	*−2.6*	*(//)*	*(//)*
**Boel et al. (2016)**	subthalamic nucleus = 65	Linear mixed model	COWAT Phonemic fluency b	50	41.2	49.6	41.2	NS
				*−12*	*−13.9*	*−10.1*	*−12.8*	*P* = 0.35
	GPi = 63	Linear mixed model	COWAT Semantic fluency b					
				49.8	41.4	50	42.7	NS
				*−9*	*−10.5*	*−8.1*	*−10.7*	*P* = 0.28
							
							
**Follet et al. (2010)**	subthalamic nucleus = 147	ANOVA	Phonemic fluency b	44.9	39	46.6	41.8	*NS*
				*−12.1*	*−12*	*−12*	*−11.9*	*P* = 0.33
	GPi = 152							
		ANOVA	Semantic fluency b	47	41.2	50.4	44.7	*NS*
				*−12.4*	*−13.2*	*−10.6*	*−12.4*	*P* = 0.99
							
			WAIS working memory index b					
		ANOVA		99.3	94.1	100.8	*97*	*NS*
				*−13.7*	*−15.3*	*−13*	*−13.4*	*P* = 0.27
**Hansen et al. (2017)**	subthalamic nucleus = 17	Wilcoxon rank-sum	Boston Naming Test b	53.94	53.88 *(5.57)*	52.25	52.58 *(7.63)*	NS
				*−5.74*		*−9.43*		*P* = 0.72
	GPi = 12							
		Wilcoxon rank-sum	WAIS working memory index b					
				97.76	91	99	99.17	NS
				*−13.02*	*−11.91*	*−16.66*	*−0.91*	*P* = 0.17
							
**John et al. (2021)**	subthalamic nucleus = 23	ANOVA	Phonemic fluency b	10	8	*9*	8	NS
								*P* = 0.90
	GPi = 17							
		ANOVA	Semantic fluency b	11	7	10	7	NS
								*P* = 0.90
							
		ANOVA	Action fluency b	9	6	8	6	NS
								*P* = 0.90
							
**Okun et al. (2009)**	subthalamic nucleus = 22	Hotelling's T^2^	Phonemic fluency	0.3	−2.6	−5.6	0.3	*NS*
				*−10.7*	*−9.3*	*−6.7*	*−10.7*	*P* = 0.03
	GPi = 23	Hotelling's T^2^						
			Semantic fluency b	0.7	0.7	1.2	1.2	NS
				*−5.5*	*−5.5*	*−6.3*	*−6.3*	*P* = 0.57
							
**Rothlind et al. (2006)**	subthalamic nucleus = 19	ANOVA	Phonemic fluency b	40.3	36.5^31.5>	36	29.70^32.90>	NS
				*−12.1*	*(12.1) (14.2)*	*−14*	*(11.9) (10.1)*	*P* = 0.19
	GPi = 23							
		ANOVA	Semantic fluency b	16.9	17.3^15.2>	19.2	19.1^16.70>	NS
				*−4.1*	*(4.5) (4.3)*	*−3.7*	*(4.4) (3.9)*	*P* = 0.21
							
		ANOVA	WAIS −R DSST b	37.9	34.7^30.1>	35	38.20^36.80>	NS
				*−14.7*	*(10.5) (17.6)*	*−12.3*	*(11.4) (13.1)*	*P* = 0.12
							
		ANOVA	WAIS-R backwards b	6.3	5.6^6.0>	6.3	5.7^5.1>	NS
				*−1.4*	*(1.5) (1.5)*	*−1.9*	*(1.5) (0.9)*	*P* = 0.10
**Weaver et al. (2012)**	subthalamic nucleus = 70	Fisher exact tests	Phonemic fluency b	44	37.3	47.80 *(11.6)*	42.3	NS
				*−12.5*	*−14.4*		*−12.6*	*P* = 0.27
	GPi = 89	Fisher exact tests						
			Semantic fluency b	45.6	39	50.1	43.9	NS
		Fisher exact tests		*−12*	*−13.2*	*−11.4*	*−11.9*	*P* = 0.57
							
			WAIS working memory index b	97.8	91	102.2	96.6	**P = 0.06
				*−15.5*	*−17.8*	*−13.4*	*−13.9*	
							

**Abbreviations**.

*SD*, Standard Deviation. *COWAT*, Controlled Oral Word Association Test. *ANOVA*, Analysis of Variance. *WAIS-R-DSST*, Wechsler Adult Intelligence Scale Revised Digital Symbol Test. *WAIS-VCI*, Wechsler Adult Intelligence Scale Verbal Comprehension Index. *WAIS-WMI*, Wechsler Adult Intelligence Scale Working Memory Index. *WAIS-PSI*, Wechsler Adult Intelligence Scale Processing Speed Index.

** Statistically significant.

NS Not Significant.

^a^Higher score indicates worse functioning.

// Results not reported.

^Unilateral.

> Bilateral.

Note: Rothlind et al. [29] provides two sets of follow-up results split into unilateral and bilateral stimulation.

b Higher score indicates better functioning.

### 3.5 Interpretation of Findings; narrative Synthesis

The findings on both verbal fluency and working memory present a complex picture of how DBS in the subthalamic nucleus and GPi influence cognitive functions in PD. The variations across studies, and even within specific areas of these cognitive functions, underscore the intricate interplay between these areas.

### 3.6 Verbal fluency

The observed trend of a decline in verbal fluency scores across nearly every study underscores the potential cognitive impacts of DBS on both subthalamic nucleus and GPi. Table 4 visually depicts such findings. While Boel et al. [27] reported minimal change in verbal fluency scores for GPi, a distinct decrease was noted for subthalamic nucleus. Similarly, Hansen et al. [26] observed minimal changes for both subthalamic nucleus and GPi. This variation suggests that while the overarching trend indicates a decline in verbal fluency, specific factors inherent to each study or treatment protocol may modulate the severity and nature of a decline.

**Table 4:** Collated findings for verbal fluency.

**Paper**	**verbal fluency outcome subthalamic nucleus (↑ improvement, ↓ decrease, —minimal change)**	**verbal fluency outcome GPi (↑ improvement, ↓ decrease)**	**Between group statistical significance (—nonsignificant, + significant)**
**Alley (2022)**	↓	↓	**—**
**Boel et al. (2016)**	↓	**—**	**—**
**Follet et al. (2010)**	↓	↓	**—**
**Hansen et al. (2017)**	**—**	**—**	**—**
**John et al. (2021)**	↓	↓	**—**
**Okun et al. (2009)**	↓	↓	**—**
**Rothlind et al. (2006)**	↓	↓	**—**
**Weaver et al. (2012)**	↓	↓	**—**

The study by Alley [24] is particularly intriguing, showing a greater decline in subthalamic nucleus with non-significant overall verbal fluency outcomes. Alley [24] found a greater decline in verbal fluency for participants with subthalamic nucleus DBS, although the results were not statistically significant. This absence of significance could be attributed to the small sample size, limiting its statistical power to detect true differences. While the trend observed by Alley [24] in their small sample hints at a potential decline in verbal fluency for patients under subthalamic nucleus DBS, it's important to interpret this finding with caution. Larger trials have indicated no significant difference between subthalamic nucleus and GPi DBS [27].

In contrast, Boel et al. [27] conducted a RCT with a considerably larger sample size (n = 128) in the Netherlands, finding an equal decline in both GPi and subthalamic nucleus DBS, indicating a lack of difference between the two brain regions. This finding aligns with Rothlind et al. [29], further affirming the notion that the choice between GPi and subthalamic nucleus may not significantly impact verbal fluency outcomes. Conducting a RCT in the USA involving 42 participants, Rothlind et al. [29] observed an equal decline in both GPi and subthalamic nucleus DBS patients. Further suggesting that GPi and subthalamic nucleus may not significantly impact verbal fluency outcomes. So, the findings may suggest a commonality in the underlying mechanisms affected by the interventions.

John et al. [25] added further complexity to the collated findings by showing a rapid decline in phonemic and semantic fluency, but not action fluency. John et al. [25] conducted a NRCT in the USA, involving 40 participants. Their findings were particularly interesting, with prominent declines for phonemic and semantic verbal fluency, but not for action fluency. This selectivity in the domains of verbal fluency that were impacted provides valuable insights into the complex nature of the changes that may follow DBS. Moreover, they observed an equal decline in verbal fluency in subthalamic nucleus and GPi, consistent with some of the other studies in the review [27,29]. The specificity of the verbal fluency domains that were affected suggests that the cognitive changes associated with DBS may be more intricate and domain-specific than previously assumed. Illustrating the heterogeneous effects that these interventions can have on different cognitive processes, perhaps linked to the underlying neural pathways that are modulated by the stimulation.

Moreover, further inconsistencies in research findings were evident in the paper by Okun et al. [3]. Performing aRCT in the USA, including 52 participants, Okun et al. [3] found worse performance on letter fluency tasks following subthalamic nucleus DBS, but performance on the semantic fluency task remained steady from baseline to follow up. Thus, potentially indicating a more complex and selective impact of DBS on cognitive processes within the broader domain of verbal fluency. This study contradicts the observations by Boel et al. [27] and Rothlind et al [29] that found an equal decline.

### 3.7 Working memory

The varied findings concerning the impact of subthalamic nucleus and GPi DBS on working memory in PD patients underscore the nuanced nature of this relationship. When examining these studies collectively, it becomes evident that certain patterns and potential contradictions emerge, offering valuable insights. Table 5 visually displays this.

**Table 5:** Collated findings for working memory.

**Paper**	**working memory outcome subthalamic nucleus (↑ improvement, ↓ decrease, — minimal change)**	**working memory outcome GPi (↑ improvement, ↓ decrease, — minimal change)**	**Between group statistical significance (—nonsignificant, + significant)**
Follet et al. (2010)	↓	**—**	**—**
Hansen et al. (2017)	**—**	**—**	**—**
Weaver et al. (2012)	↓	↓	+

The results produced by Follet et al [28] favour GPi DBS concerning working memory outcomes, suggesting a lessened cognitive decline for patients receiving GPi DBS compared to those under subthalamic nucleus DBS. Such findings contribute significantly to the current debate on the optimal DBS target for PD patients, especially when considering the cognitive repercussions. Weaver et al. [30] further deepened this discourse. Their study revealed decreases in working memory for both subthalamic nucleus and GPi. This nuanced insight aligns with their larger narrative, where they introduced a layer of complexity by revealing that cognitive responses post-DBS evolve over time. While the GPi group exhibited an initial decline in working memory that later stabilised, the subthalamic nucleus group’s decline manifested notably later, specifically at 36 months post-intervention with significant interactions between subthalamic nucleus and GPi, favouring the latter. Such findings not only emphasise the difference in DBS sites, but also underscore the dynamic nature of working memory outcomes following DBS. This unfolding pattern of cognitive response could be rooted in the neuroplastic capabilities of the brain, which may be differentially modulated by GPi and subthalamic nucleus stimulations, as postulated by studies like Petzinger et al. [31].

On the other hand, Hansen et al. [26] offers more cautionary findings. Given its retrospective design and a relatively smaller sample size, the study didn't find clear-cut working memory differences between GPi and subthalamic nucleus. Yet, the trends they identified show the diverse responses individuals might have to DBS interventions. Such nuances suggest that comprehensive patterns might be clearer in larger-scale studies, thereby emphasising the value of diverse methodological approaches to encapsulate the broad spectrum of patient experiences.

## 4 Discussion

This systematic review found mixed evidence on the effects of DBS in PD in relation to verbal fluency and working memory. Although one study found a statistically significant working memory benefit for GPi-DBS, the overall findings were inconsistent across targets. Verbal fluency declines were commonly reported, but not consistently linked to either the subthalamic nucleus or GPi.

These findings challenge the widespread view that subthalamic nucleus DBS poses greater cognitive risks than GPi. While early studies and clinical guidelines often urge a cautious approach to stimulation in the subthalamic nucleus due to concerns about verbal fluency decline [32], this review found no consistent evidence to support this distinction. Both targets were associated with verbal fluency declines, but the magnitude and direction of effects were variable and not systematically attributable to one target. This suggests that DBS target selection should be guided by a more nuanced, patient-centred framework. Personalisation might consider factors such as the patient’s baseline cognitive functioning, degree of executive dysfunction, occupational or social reliance on verbal skills, and the presence of pre-existing psychiatric or language-related conditions [33]. In addition, age, disease duration, and treatment goals may all influence the most appropriate target [[33], [34], [35]]. Rather than assuming GPi stimulation is inherently safer cognitively, clinicians should engage patients in shared decision-making that weighs motor and non-motor trade-offs according to individual circumstances.

The variability in verbal fluency findings may reflect differences in attentional demands and executive functioning [36,37]. Gadot et al. [38] suggest that attentional networks modulate verbal fluency, and differential effects on these networks by DBS, depending on electrode placement or stimulation parameters, might account for the variability in findings. Elgebaly [39] emphasised working memory's dynamic nature post-DBS. The lack of significant differences in some studies, like Hansen et al. [26], suggests that further research is needed to identify factors that might influence working memory outcomes. Literature shows working memory is closely linked with attention and executive functions, and deficits in attention can compromise working memory performance [40]. Factors such as individual disease trajectory, baseline cognition, comorbidities, and surgical precision, especially electrode placement, have all been shown to influence cognitive trajectories post-DBS [[41], [42], [43], [44], [45], [46]]. These findings support the need for personalised approaches that go beyond target selection alone.

One critical area for future exploration is the distinction between unilateral and bilateral stimulation in subthalamic nucleus and GPi DBS, and its effects on verbal fluency and working memory [29]. Future research must carefully consider these distinctions, specifically investigating how either approach impacts phonemic and semantic fluency. Del Bene et al. [47] found that unilateral stimulation of the right hemisphere was associated with less decline in verbal fluency compared to left hemisphere or bilateral DBS, suggesting that verbal fluency deficits may be at least partially mediated by disruption of dominant-hemisphere language networks. These findings support the emerging view that hemispheric targeting matters, and that unilateral or staged bilateral approaches may be preferable for patients at higher risk of cognitive decline. Insights from lesion-based interventions, such as radiofrequency ablation, pallidotomy, and focused ultrasound thalamotomy, have identified similar effects on cognitive outcomes [48]. These studies underscore the role of hemispheric specialisation and should be considered when planning DBS interventions, particularly in cognitively vulnerable patients. Another promising avenue for personalising DBS involves adjusting stimulation parameters, particularly frequency, to mitigate cognitive side effects. While traditional DBS protocols often use high-frequency stimulation to optimise motor benefits, studies have shown that lower-frequencies may reduce cognitive disruptions, without compromising motor outcomes. Grover et al. [49] and Lee et al. [50] both reported that low-frequency subthalamic nucleus stimulation resulted in improved speech intelligibility and preserved verbal fluency compared to higher-frequency protocols. Similarly, Wojtecki et al. [51] demonstrated frequency-dependent trade-offs between cognitive and motor functions, suggesting that fine-tuning stimulation parameters could yield more balanced outcomes. Although the current evidence base is small, these findings offer a potential method for tailoring DBS to individual cognitive profiles.

The review highlights several methodological limitations, including the use of diverse instruments across studies, which complicates direct comparisons. Variations in follow-up durations and geographical diversity also contribute to the observed heterogeneity in results. Additionally, differences in stimulation sites and the lack of longitudinal analysis limit the understanding of DBS's long-term cognitive effects. These limitations underscore the need for more standardised and comprehensive approaches in future research. One significant limitation of this systematic review is that it was conducted by a single researcher. While this does not inherently undermine the validity or rigor of the research, it can introduce challenges [52]. The process of selecting studies, evaluating their quality, extracting data, and synthesising findings ideally benefits from collaboration [53]. Having multiple researchers involved can mitigate individual biases and provide different perspectives on interpretations [54]. Moreover, while *meta*-analysis could have offered a quantitative summary of findings, significant variability in the methodological approaches, cognitive measures, and follow-up durations of the included studies made it impractical. By adopting a narrative synthesis, this review was able to highlight nuanced trends and contextual factors that might otherwise be obscured in a *meta*-analytic approach. Despite its limitations, this review's strengths lie in its rigorous methodology and inclusion of studies with diverse methodologies. Additionally, the review's attention to cultural and geographical contexts adds depth to its findings, ensuring that the results are culturally informed and nuanced.

## 5 Conclusion

While no clear pattern emerged linking target site to consistent verbal fluency outcomes, and only one study showed a significant working memory benefit favouring GPi stimulation , the overall evidence highlights the need for a more personalised approach to DBS. Factors such as individual cognitive profiles, stimulation laterality, and frequency parameters appear to play an important role in shaping outcomes. Future research should prioritise these variables to better inform target selection and optimise both motor and cognitive results, ultimately enhancing patient-centred care and quality of life.

## CRediT authorship contribution statement

**Hannah Trotman:** Writing – original draft, Visualization, Validation, Project administration, Methodology, Investigation, Formal analysis, Conceptualization. **Benjamin Jelley:** Writing – review & editing, Supervision, Project administration, Conceptualization. **Katja Umla-Runge:** Writing – review & editing, Supervision, Project administration, Conceptualization.

## Declaration of competing interest

The authors declare that they have no known competing financial interests or personal relationships that could have appeared to influence the work reported in this paper.

## Figures and Tables

**Table:** 

	*Keywords (Ti, Ab)*	*EMBASE*	*MEDLINE*	*EMCARE*	*PSYCINFO*	
*1*	Deep brain stimulation	20718	11659	1824	4631	
*2*	DBS	20665	9111	1463	3144	
*3*	Neurostimulation	4956	2174	550	841	
*4*	Brain stimulation	28269	15131	2803	9832	
*5*	Subthalamic nucleus	9784	4651	1019	1948	
*6*	subthalamic nucleus	7129	3201	578	1309	
*7*	Subthalamic region	108	72	7	32	
*8*	Globus pallidus	12430	4392	1501	1703	
*9*	Pallidus	12594	4468	1517	1735	
*10*	GPi	7761	10921	1165	590	
*11*	Verbal fluency	9658	5026	1595	10105	
*12*	Speech	49407	15574	20618	15084	
*13*	Oral communication	815	299	246	15356	
*14*	Speech production	3893	6935	1466	5055	
*15*	Linguistics	18404	6970	4456	19124	
*16*	Parkinsons disease	106025	58628	11545	23561	
*17*	Parkinsons	106025	58628	11545	23561	
*18*	Parkinsonism	106025	58628	11545	23561	
*19*	1 OR 2 OR 3 OR 4	38483	19399	3654	10526	
*20*	5 OR 6 OR 7	11561	5173	1112	2024	
*21*	8 OR 9 OR 10	18299	14475	1837	1970	
*22*	11 OR 12 OR 13 OR 14 OR 15	77078	30839	26736	45905	
*23*	16 OR 17 OR 18	106025	58628	11545	23561	
	19 AND 20 AND 21 AND 22 AND 23	**74**	**20**	**8**	**11**	
