# Supplementary material for: Comparative effects of deep brain stimulation in subthalamic nucleus and globus pallidus interna on verbal fluency and working memory in adult populations with parkinson’s disease: A systematic review
Source: Clin Park Relat Disord. 2025 Jun 9;13:100355. doi: 10.1016/j.prdoa.2025.100355 (PMC12205537; doi:10.1016/j.prdoa.2025.100355)
Supplement: Supplementary Data 1 [file mmc1.docx]

Supplemental materials.

| **Study** | **Design** | **Country** | **Average age (years)** | | **Gender (male:female)** | | **Subgroups N=** | **N=** | **Verbal fluency measure** | **Working memory measure** | **Results** | **Statistical test outcome** |
| --- | --- | --- | --- | --- | --- | --- | --- | --- | --- | --- | --- | --- |
|  |  |  | **GPi** | **subthalamic nucleus** | **GPi** | **subthalamic nucleus** |  |  |  |  |  |  |

Supplemental materials 1. Blank data extraction form
